# FMO-4 SLOWS AGING DOWNSTREAM OF MTOR AND DR THROUGH ER TO MITOCHONDRIA CALCIUM SIGNALING

**DOI:** 10.1093/geroni/igae098.1719

**Published:** 2024-12-31

**Authors:** Angela Tuckowski, Safa Beydoun, Elizabeth Kitto, Ajay Bhat, Marshall Howington, Aditya Sridhar, Mira Bhandari, Kelly Chambers, Scott Leiser

**Affiliations:** University of Michigan, Michigan, United States; University of Michigan, Ann Arbor, Michigan, United States; University of Michigan, Ann Arbor, Michigan, United States; University of Michigan, University of Michigan, Michigan, United States; University of Michigan, Ann Arbor, Michigan, United States; University of Michigan, Michigan, United States; University of Michigan, Michigan, United States; University of Michigan, Michigan, United States; University of Michigan, Ann Arbor, Michigan, United States

## Abstract

The geroscience field has identified several pathways that regulate aging across species. Of these pathways, dietary restriction and the reduced mechanistic target of rapamycin (mTOR) signaling are among the best studied but still not fully understood interventions. Recent work from our lab and others established the flavin containing monooxygenase family of enzymes as regulators of longevity across species. While others found that Fmos are induced by longevity interventions in mice, we found that Cefmo-2 promotes longevity, stress resistance, and healthspan by rewiring endogenous metabolism. These results suggest that the FMO family could regulate longevity in multiple species, including humans. Our new work focuses on the role of C. elegans FMO-4, which is structurally similar to FMO-2 but expressed in a different tissue. Our data show that fmo-4 confers stress resistance and is required for DR- and mTOR-mediated longevity. We also find that fmo-4 does not act similar to fmo-2 but downstream of it, and interacts with calcium signaling and stress response to promote healthy aging. Under the hypothesis that modified calcium signaling is crucial for ER and mitochondrial homeostasis, we find evidence of 1) the metabolic, stress, and aging processes in which FMO-4 activity plays a role, and 2) the likely mechanisms for these roles. Overall, our data highlight the importance of FMOs in longevity, and reveal a novel mechanism for FMO regulation of longevity and metabolism.

